# Epidemiology and patterns of hypertension in semi-urban communities, south-western Nigeria

**DOI:** 10.5830/CVJA-2016-037

**Published:** 2016

**Authors:** MA Olamoyegun,, SO Iwuala, SO Asaolu, R Oluyombo

**Affiliations:** Ladoke Akintola University of Technology (LAUTE CH), LAUTECH Teaching Hospital, Ogbomoso, Nigeria; Department of Medicine, Endocrinology, Diabetes and Metabolism Unit, Lagos University Teaching Hospital, Idi Araba, Lagos, Nigeria; Department of Internal Medicine, LAUTECH Teaching Hospital, Ogbomoso, Nigeria; Department of Medicine, Federal Teaching Hospital, Ido Ekiti, Nigeria

**Keywords:** epidemiology, hypertension, pattern, semi-urban

## Abstract

**Objective:**

To determine the prevalence and subtypes of hypertension among semi-urban residents in south-western Nigeria.

**Methods:**

All adults aged 18 years or older in 10 semi-urban communities were recruited for the study. The blood pressure (BP) reading taken with a validated electronic BP monitor after at least 10 minutes of rest was used in the analysis. Hypertension was defined as BP ≥ 140/90 mmHg.

**Results:**

Seven hundred and fifty subjects with a mean age of 61.7 ± 18.2 years participated in the study. The prevalence of hypertension was 55.5%. Stage 2 hypertension was the most common, present among 225 (54.1%) of the participants with hypertension, and 191 (45.9%) had stage 1 hypertension. Of those with hypertension, systolic–diastolic hypertension (SDH) was present among 198/416 (47.6%), while isolated systolic hypertension (ISH) and isolated diastolic hypertension (IDH) were present among 181/416 (43.6%) and 37/416 (8.9%), respectively. The prevalence of hypertension increased significantly with age.

**Conclusion:**

The prevalence of hypertension was high in these semi-urban communities. Hence, increased awareness and integrating hypertension care into primary healthcare and other community health services in these areas may prove beneficial in ameliorating its adverse effects.

## Objective

Hypertension is regarded as a major public health problem^1^ and is also an important threat to the health of adults in sub-Saharan Africa.^2^ Emerging evidence identifies hypertension as a major cause of morbidity and mortality globally, including sub-Saharan Africa.^2^-^4^ Hypertension is now recognised as one of the most important causes of cardiovascular diseases, accounting for almost 40% of the diseases on the African continent, including Nigeria.^5^,^6^

According to a World Health Organisation report, hypertension is the third cause of deaths, accounting for one in eight deaths worldwide.^7^ The overall worldwide burden of hypertension in the year 2000 was 26.4% of the adult world population, 34.3% in developed and 65.8% in developing countries.^8^ Also, about 62% of cardiovascular diseases (CVDs) and 49% of ischaemic heart disease (IHD) are attributable to suboptimal blood pressure (systolic blood pressure > 115 mmHg).^9^ Indeed, it has been projected that up to three quarters of the world’s hypertensive population will be in economically developing countries by the year 2025.^10^ Nigeria, due to its population size and the projected increase in the prevalence of hypertension, will face a huge economic health burden from hypertension.

The prevalence of hypertension has been variously studied in Nigeria, however, most studies were done among urban and rural dwellers. In a review of hypertension prevalence in Nigeria by Akinlua et al.^11^ using a blood pressure cut-off value of 140/90 mmHg, the crude prevalence of hypertension ranged from 6.2 to 48.9% and 10.0 to 47.3% for males and females, respectively. Comparing urban versus rural differences in crude prevalence, estimates showed an overall prevalence ranging from 17.5 to 51.6% in urban areas and 4.6 to 43.0% in rural areas. There have been few studies done to determine the prevalence of hypertension among dwellers in semi-urban areas, which have mixtures of urban and rural areas.

Our study therefore aimed to assess the prevalence and subtypes of hypertension among the population in semi-urban communities in south-western Nigeria. The findings in this study will further add to the available data on the increasing prevalence of hypertension in Nigeria, thereby stimulating increased effort by health policy makers to control the emerging health burden. This study could also demonstrate the need for prevention and control of hypertension in daily medical practice.

## Methods

This study was conducted in Ekiti State, located in the southwestern zone of Nigeria. The state is divided into three senatorial zones (Ekiti south, north and west). The study area, Ekiti north senatorial zone, is made up of five local government areas (Ido/Osi, Ikole, Moba, Ilejemeje and Oye). A total of 10 communities (all semi-urban) were randomly selected within the senatorial district (two communities per local government area).

A semi-urban community is one with a population of between 500 and 5 000, with basic amenities such as secondary and primary schools, electricity and a few primary healthcare (PHC) centres, with few private clinics.^12^ In a semi-urban community, most inhabitants are individuals with low socio-economic status, mainly artisans, traders and low-income workers who live in over-crowded areas with poor sanitary conditions. There is an adaption to a Western lifestyle in semi-urban communities compared to a rural community.

This was a cross-sectional, community-based study in which 750 participants were recruited. At the community level, screening of residents who volunteered to participate was undertaken by trained interviewers until approximately equal numbers of participants were selected from each of the selected sites. A person not normally resident in the community was not included in the analysis (even if screened).

Community approval and entry was facilitated after interacting with the heads of these communities, religious leaders and other community leaders and also by meeting with the health workers of PHCs available in these communities. The purpose of such meetings was to explain the aims of the study and obtain communal consent. The study was approved by the institutional ethics committee of the Federal Medical Centre, Ido-Ekiti.

A questionnaire, which was researcher-developed and interviewer-administered, was used to obtain data from the participants. The questionnaire contained two parts, the first being demographic information including age, gender, occupation, monthly income and family history of hypertension. The second part involved measurement of height, weight, waist and hip circumferences, and blood pressure.

Height was measured without shoes or headgear, using a wooden platform stadiometer ruled to the nearest 0.5 cm, while weight was measured to the nearest 0.5 kg using a weighing scale (Hanson HX5000 electronic bathroom scale). Body mass index (BMI) was calculated as weight (kg) divided by the square of the height in metres (m^2^). Waist circumference (WC) was measured to the nearest 0.1 cm, at the midpoint between the costal margin and iliac crest, at the end of normal expiration, using a non-stretchable measuring tape. Hip circumference (HC) was measured at the level of the greater trochanters (widest diameter of hips) to the nearest 0.1 cm with a measuring tape, while the subject was standing with the arms by the side and feet together.^13^,^14^ The waist-to-hip ratio (WHR) was calculated from WC:HC

Blood pressure was measured on the left arm in a seated position with the subjects in a relaxed state, using a validated electronic blood pressure monitor (Omron MX2 Basic, Omron Healthcare Co, Ltd, UK). A standard aneroid sphygmomanometer with an adult cuff size (Medicare instrument, NUXI, Ltd, China) was used to confirm the reading by electronic monitor. Blood pressure was classified according to the seventh Joint National Committee and Treatment of High Blood Pressure (JNC7)^15^ criteria into normal, prehypertension, stage 1 hypertension and stage 2 hypertension. Hypertension was defined as systolic blood pressure (SBP) ≥ 140 mmHg and/or diastolic blood pressure (DBP) ≥ 90 mmHg and/or concomitant use of antihypertensive medications.^16^

## Statistical analysis

The Statistical Package for Social Sciences software version 17 (SPSS Inc, Chicago, IL) was used for data analysis. Continuous variables are expressed as means ± standard deviation (SD) while categorical variables are presented as frequencies and percentages. Comparison for statistical significance was done with the Student’s t-test for continuous variables that were normally distributed, or Chi-square analysis for categorical variables. All tests were two-tailed with p < 0.05 taken as statistical significance.

## Results

A total of 856 participants were encountered for the study but only 750 participants had complete data for analysis, which represented a response rate of 87.6%. The majority were females (529, 70.5%). The mean age of the participants was 61.7 ± 18.2 years. Farmers and petty traders dominated the occupation of the participants (520, 68.4%). With regard to educational level, the majority (77.6%) had either no formal education or primary school as the highest educational level attained. Onlysix (0.8%) persons were current smokers. The sociodemographic characteristics of the subjects are shown in Table 1. Mean BMI, WC, WHR, SBP and DBP of the study participants were 23.4 ± 5.5 kg/m^2^, 85.7 ± 11.9 cm, 0.93 ± 0.36, 142.4 ± 28.6 mmHg and 81.6 ± 14.2 mmHg, respectively.

**Table 1:** Sociodemographic and clinical characteristics of the study population

**	*All*	*Male*	*Female*	**
*Variable*	*(n = 750)*	*(n = 218)*	*(n = 542)*	*p-value*
Age (years)				
< 20	5 (0.7)	3 (60.0)	2 (40.0)	0.072
20-40	112 (14.9)	41 (36.9)	70 (63.1)	
41–60	212 (28.3)	53 (25.6)	154 (74.4)	
> 60	421 (56.1)	120 (29.1)	293 (70.9)	
Mean age ± SD	61.7 ± 18.2	60.0 ± 20.0	62.4 ± 17.3	0.090
Marital status				
Single	45 (6.0)	31 (68.9)	14 (31.1)	< 0.001
Married	434 (57.9)	177 (40.8)	257 (59.2)	
Widow/widower	268 (35.7)	11 (4.1)	257 (95.9)	
Divorced	3 (0.4)	2 (66.7)	1 (33.3)	
Occupation				
Unemployed	85 (11.3)	14 (16.5)	71 (83.5)	< 0.001
Petty trader	309 (41.2)	9 (2.9)	300 (97.1)	
Farmer	211 (28.1)	111 (52.6)	100 (47.4)	
Unskilled labourer	52 (6.9)	35 (67.3)	17 (32.7)	
Clerk/typist	1 (0.1)	1 (100.0)	0 (0.0)	
Professional	40 (5.3)	16 (40.0)	24 (60.0)	
Other	52 (6.9)	35 (67.3)	17 (32.7)	
Educational level				
None	413 (55.1)	89 (21.5)	324 (78.5)	< 0.001
Primary	169 (22.5)	54 (32.0)	115 (68.0)	
Secondary	107 (14.3)	52 (48.6)	55 (51.4)	
Tertiary	61 (8.1)	26 (42.6)	35 (57.4)	
Income (Naira)				
< 20 000	626 (83.5)	159 (25.4)	467 (74.6)	< 0.001
20 000–40 000	89 (11.9)	40 (44.9)	49 (55.1)	
41 000–60 000	26 (3.5)	16 (61.5)	10 (38.5)	
61 000–100 000	6 (0.8)	5 (83.3)	1 (16.7)	
> 100 000	3 (0.4)	1 (33.3)	2 (66.7)	
BMI (kg/m^2^)				
< 25	525 (70.0)	172 (77.8)	353 (66.7)	< 0.001
25–29.9	161 (21.5)	44 (19.9)	117 (22.1)	
≥ 30	64 (8.5)	5 (2.3)	59 (11.2)	
Mean BMI (kg/m^2^)	23.4 (5.5)	22.6 (5.5)	23.7 (5.5)	0.015
Mean WC (cm)	85.7 (11.9)	83.3 (9.6)	86.7 (12.7)	7lt; 0.001
Mean WHR	0.93 (0.36)	0.93 (0.06)	0.93 (0.43)	0.919
Mean SBP (mmHg)	142.4 (28.6)	142.3 (28.7)	142.4 (28.5)	0.976
Mean DBP (mmHg)	81.6 (14.2)	81.1 (15.0)	81.8 (13.8)	0.534

Fisher’s exact, values are mean (SD) or n (%).

**Fig. 1.**
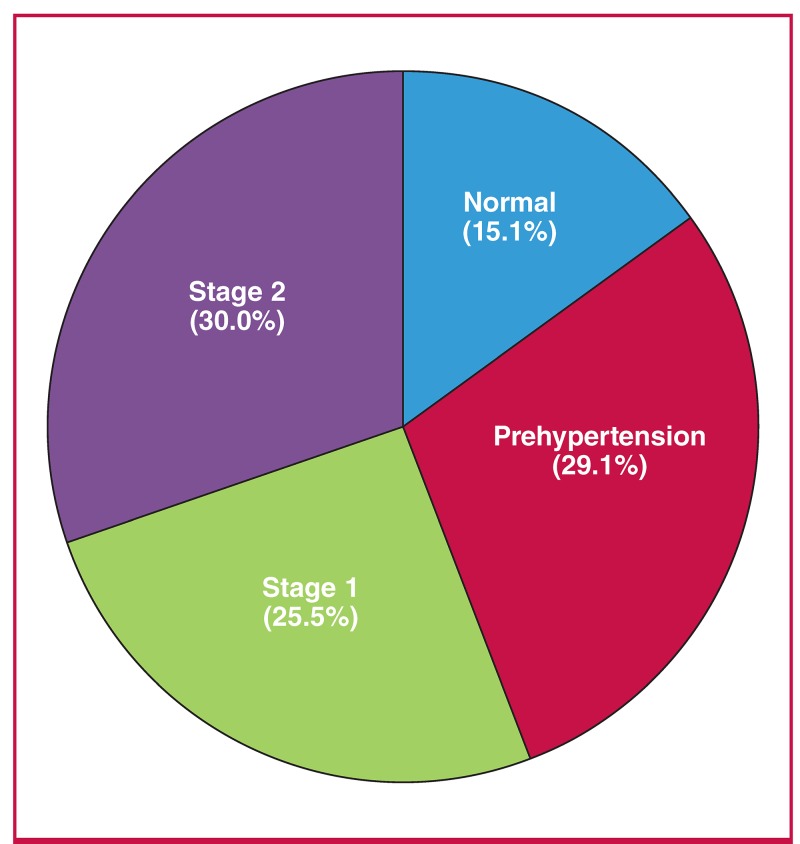
Prevalence of hypertension according to JNC7.

Out of the 750 participants, 116 (15.5%) had normal blood pressure, 218 (29.0%) had prehypertension and 416 (55.5%) had hypertension. Of the study participants, 195 (26.0%) had been diagnosed with hypertension previously, 81 (10.9%) were on antihypertensive medication, and 24 (3.2%) had a positive family history of hypertension. Twenty (2.6%) said they were taking herbal remedies for hypertension. The proportion of those on antihypertensive medication who had good blood pressure control of ≤ 140/90 mmHg was 22.0%. Of the participants with hypertension, isolated systolic hypertension (ISH) was present among 181/416 (43.6%) while isolated diastolic hypertension (IDH) and systolic–diastolic hypertension (SDH) were present among 37 (8.9%) and 198 (47.6%), respectively (Fig. 1).

**Fig. 2.**
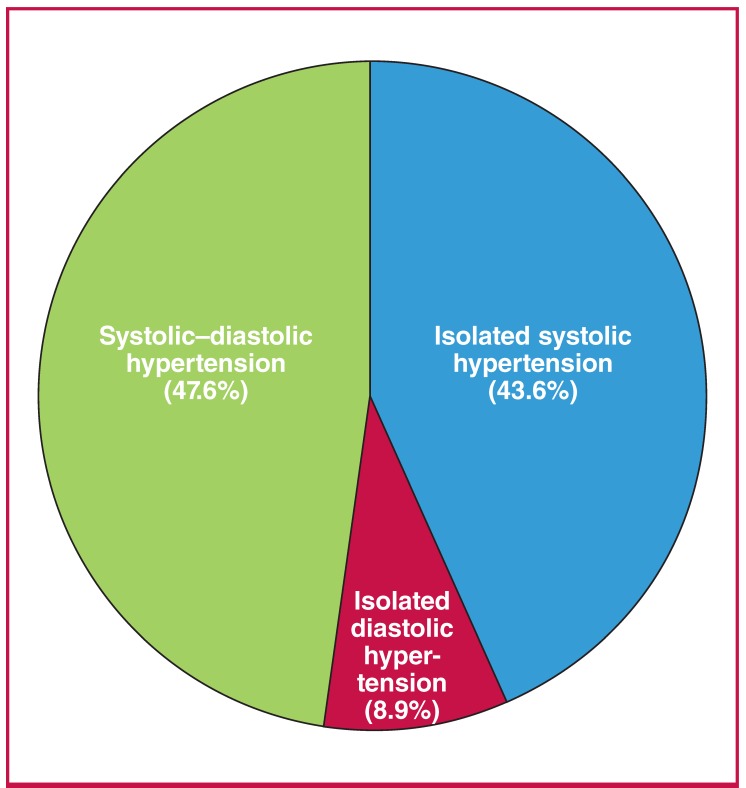
Prevalence of hypertension according to subtypes.

Stage 2 hypertension was the most common, being present among 225 (54.1%) of the participants with hypertension. Of those with hypertension 191 (45.9%) had stage 1 hypertension (Fig. 2). Table 3 shows the gender and sociodemographic relationships of the hypertension subtypes. ISH (67.4%) and IDH (59.5%) were more prevalent among females compared to males. Also, participants with ISH were significantly older than those with IDH (p = 0.014). After adjusting for confounding variables, factors associated with hypertension among the study participants were age group, educational level and body mass index (BMI) (Table 2).

**Table 2:** Predictors of hypertension among the study participants

**	*No hypertension*	*Hypertension*	**
*Variable*	*(n = 334)*	*(n = 416)*	*p-value*
Gender			
Male	96 (28.7)	125 (30.0)	0.697
Female	238 (71.3)	291 (70.0)	
Age (years)			
< 20	4 (1.2)	1 (0.2)	< 0.001
20–40	82 (24.6)	30 (7.2)	
41–60	98 (29.3)	114 (27.4)	
> 60	150 (44.9)	271 (65.1)	
Marital status			
Single	25 (7.5)	20 (4.8)	0.084
Married/widow/widower/divorced	309 (92.5)	396 (95.2)	
Educational level			
None	152 (45.5)	261 (62.7)	< 0.001
Primary	84 (25.1)	85 (20.4)	
Secondary	62 (18.6)	45 (10.8)	
Tertiary	36 (10.8)	25 (6.0)	
Average family income (Naira)			
< 20 000	272 (81.4)	354 (85.1)	0.472
20 000–40 000	43 (12.9)	46 (11.1)	
41 000–60 000	15 (4.5)	11 (2.6)	
61 000–100 000	2 (0.6)	4 (1.0)	
> 100 000	2 (0.6)	1 (0.2)	
BMI (kg/m^2^)			
< 25	172 (77.8)	353 (66.7)	< 0.001
25–29.9	44 (19.9)	117 (22.1)	
≥ 30	5 (2.3)	59 (11.2)	

**Table 3:** Comparison of sociodemographic and clinical characteristics according to hypertension subtypes

**	*Both*	*IDH*	*ISH*	**
*Variable*	*(n = 198)*	*(n = 37)*	*(n = 181)*	*p-value*
Age (years)	68.2 (13.7)	61.4 (18.4)	64.4 (17.6)	0.014
Gender				
Male, n (%)	51 (25.8)	15 (40.5)	59 (32.6)	0.117
Female, n (%)	147 (74.2)	22 (59.5)	122 (67.4)	
Educational level				
None	133 (67.2)	18 (48.6)	110 (60.8)	0.199
Primary	38 (19.2)	10 (27.0)	37 (20.4)	
Secondary	17 (8.6)	4 (10.8)	24 (13.3)	
Tertiary	10 (5.1)	5 (13.5)	10 (5.5)	
Income (Naira)				
< 20 000	174 (87.9)	29 (78.4)	151 (83.4)	0.512
20 000–40 000	18 (9.1)	7 (18.9)	21 (11.6)	
41 000–60 000	4 (2.0)	1 (2.7)	6 (3.3)	
61 000–100 000	1 (0.5)	0 (0.0)	3 (1.7)	
> 100 000	1 (0.5)	0 (0.0)	0 (0.0)	
Mean BMI (kg/m^2^)	23.4 (5.3)	24.1 (5.1)	23.7 (7.3)	0.823
Mean WC (cm)	87.3 (11.2)	88.9 (10.5)	86.5 (12.7)	0.483
Mean WHR	0.94 (0.15)	0.95 (0.05)	0.92 (0.07)	0.109
Mean SBP (mmHg)	169.8 (25.3)	125.5 (9.1)	158.3 (17.2)	< 0.001
Mean DBP (mmHg)	98.9 (9.9)	92.4 (4.4)	78.6 (7.8)	< 0.001

### Discussion

This study assessed the prevalence and pattern of hypertension in its different subtypes in semi-urban communities in southwestern Nigeria. The majority of the participants were females, as is common in most cross-sectional studies.

Married petty traders made up the majority of the population, which was not unusual, as most people living in semi-urban areas participate in business and petty trading. Slightly more than half of the population had no formal education, limiting their access to quality information about healthy lifestyles. Although the government is putting efforts into increasing awareness in these rural and semi-urban settlements,^17^,^18^ health education is still largely inadequate.

Estimates from our study showed that 55.5% of the adults had hypertension, with SDH being the commonest subtype. This prevalence was much higher than the 29.7% reported by Adedoyin et al.^19^ in south-western Nigeria, and the 46.4% reported by Ejim et al.^20^ in south-eastern Nigeria. This higher prevalence may have been related to participants being older in our study compared to these other studies (61.6 vs 41.5 years and 61.6 vs 59.8 years, respectively).

Many studies have recorded a high prevalence of hypertension among elderly participants. For example, Peltzer et al.^21^ in a study among the elderly in 2008 recorded a high prevalence of hypertension (77.3%, mean age 65 years). Other studies reported higher prevalence rates of hypertension among older adult population surveys in Tanzania in 2010 (69.9%, mean age 76 years), Tunisia in 2003 (69.3%, mean age 69 years) and Senegal in 2009 (65.4%, mean age 69.5 years).^22^-^24^ Similar to Banegas et al.^25^ and Onwubere et al.,^26^ a larger percentage of participants in our study had ISH compared to IDH. ISH was also more common in those above 60 years of age, compared to IDH.

Age is known to significantly influence the prevalence and pattern of elevated blood pressure, therefore SBP tends to increase with advancing age as a result of loss of arterial compliance, while DBP tends to plateau or decrease after 50 years of age. The decrease in compliance results in higher SBP. An increase in peripheral resistance is also known to result in elevated DBP, whereas loss of elasticity in the large vessels causes a reduction with increasing age. Therefore the net effect of these opposing factors may results in a normal or near-normal DBP,^27^,^28^ depending on the predominant factor.

The prevalence of ISH obtained in this study is much higher than in a study by Tesfaye et al.^29^ Our study showed that only 26% of individuals with hypertension had been diagnosed previously, 10.9% were on antihypertensive treatment, while 22% were controlled (BP ≤ 140/90 mmHg). This finding demonstrates a high proportion of undiagnosed, untreated and poorly controlled hypertension in Nigeria, a problem that has been reported by others. For instance, good hypertension control could only be achieved in 24.2% of the patients seen in a Port Harcourt hospital.^30^ Ekwunife et al.^31^ also found only 23.7 and 17.5% of males and females, respectively, with hypertension were on antihypertensive treatment, while 5.0% of males and 17.5% of females with hypertension were controlled.

In the present study, participants with IDH were significantly younger than those with ISH. This is in keeping with a similar study done by Adeoye et al.,^32^ who found subjects with IDH to be significantly younger among the hypertensive patients in Ibadan.

The strength of this study is in its relatively moderate sample size that was spread across many communities. It however has some limitations. Many of the participants were illiterate with no recorded biodata, so the age given may not be accurate. Also, participation in this study was voluntary, which might have influenced the results.

## Conclusion

The estimate of prevalence of hypertension obtained in this study was higher than in most other studies in Nigeria, which is contrary to the existing trend of low prevalence found many years ago in semi-urban communities. The predominant patterns of hypertension observed were both SDH and ISH. The prevalence of hypertension was found to increase with age, therefore age was a significant predictor of hypertension among these subjects.

Hypertension, because of its high prevalence, deserves to be the health priority of policy makers. Therefore policy makers and other stakeholders in the health sector need to urgently institute community-based strategies towards creating awareness of hypertension, encouraging health-seeking behavioural habits, and educating people on the main risk factors such as unhealthy diet, high salt intake and sedentary lifestyles. It is important that the findings of this study prompt appropriate response at state and national levels, towards improved detection, control and management of hypertension in Nigeria.
